# FAK inhibition disrupts tumor growth, apoptosis, and transcriptional regulation in GI-NETs

**DOI:** 10.1530/EO-25-0052

**Published:** 2025-08-14

**Authors:** Lara Toffoli, Angeliki Ditsiou, Luca Triboli, Victorine Hamm, Eva Moschioni, Francesca D’Este, Teresa Gagliano

**Affiliations:** ^1^ Cancer Cell Signalling Lab, University of Udine, Udine, Italy; ^2^ Department of Medicine, University of Udine, Udine, Italy; ^3^ École de Biologie Industrielle, Cergy, France; ^4^ ASUFC, Latisana, Udine, Italy

**Keywords:** Gi-NETs, FAK, Protac

## Abstract

**Background:**

Gastrointestinal neuroendocrine tumors (GI-NETs) are rare neoplasms with limited therapeutic options and increasing clinical incidence. Focal adhesion kinase (FAK) has been implicated in oncogenic processes across various tumor types; however, its specific role in GI-NET biology remains inadequately characterized. This study investigates the impact of FAK inhibition on GI-NET cell survival, invasive potential, and gene regulation, with the aim of evaluating FAK as a therapeutic target.

**Methods:**

Human GI-NET cell lines (GOT1 and COLO320DM) were treated with Y15, a kinase inhibitor, and PROTAC-FAK (BI-0319), a degrader that abrogates both enzymatic and scaffold functions. siRNA-mediated knockdown of FAK was employed for functional validation. Assays assessing viability and apoptosis were performed in both 2D and 3D culture conditions, while invasion and colony formation were assessed in 2D culture. Western blotting, immunofluorescence, and qRT-PCR were used to evaluate molecular effects. Public transcriptomic datasets were analyzed to assess PTK2 expression across NET subtypes.

**Results:**

FAK inhibition reduced cell viability, colony formation, and invasive capacity. PROTAC-FAK, but not Y15, decreased H3K9 acetylation, indicating scaffold-dependent epigenetic modulation. On the other hand, both PROTAC-FAK and Y15 decreased H3K4 methylation levels, further supporting the role of FAK in chromatin regulation. Both compounds suppressed ERK1/2 phosphorylation and modulated RB1 expression, which was further validated by FAK knockdown. In silico analysis revealed elevated PTK2 expression in rectal and small intestinal NETs relative to pancreatic NETs.

**Conclusion:**

These findings identify FAK as a regulator of oncogenic and epigenetic pathways in GI-NETs and support its therapeutic targeting, particularly through degradation strategies that inhibit its non-catalytic functions.

**Highlights:**

- FAK inhibition impairs GI-NET viability, invasion, and colony formation in both 2D and 3D models using kinase (Y15) and PROTAC-based degraders (BI-0319).
- PROTAC-FAK uniquely reduces H3K9 acetylation, revealing a kinase-independent scaffold role for FAK in epigenetic regulation.
- esiRNA knockdown of FAK recapitulates pharmacological effects, confirming FAK as a driver of oncogenic features in GI-NET cells.
- In silico analysis identifies elevated PTK2 expression in rectal and small intestine NETs, with a strong positive correlation to RB1, supporting translational relevance.

## Introduction

Gastrointestinal neuroendocrine tumors (GI-NETs) are a rare and heterogeneous group of malignancies with a steadily rising incidence. Despite this increase, therapeutic options remain limited due to persistent resistance to available treatments and the frequent diagnosis at advanced stages of disease progression (Jensen *et al.* 2019, Garcia-Carbonero *et al.* 2023).

The molecular landscape of NETs is distinguished by an unusually low mutational burden compared to other solid tumors, such as lung or colorectal cancers. Large-scale sequencing studies of GI-NETs have consistently demonstrated a paucity of recurrent driver mutations, with only a few notable genetic alterations such as MEN1, DAXX, and ATRX mutations identified primarily in pancreatic NETs (Gagliano & Brancolini 2021, Crabtree 2022). The characteristic low mutational burden of GI-NETs suggests that epigenetic dysregulation may play a significant role in their pathogenesis; however, the biological background of these tumors is still far from being completely understood, and further investigations are mandatory to develop new therapeutic strategies (Scarpa *et al.* 2017, Di Domenico *et al.* 2017, Toffoli *et al.* 2024).

Focal adhesion kinase (FAK), encoded by the *PTK2* gene, is a non-receptor tyrosine kinase known for its central role in integrin-mediated signaling and cell adhesion (Lietha *et al.* 2007, Dawson *et al.* 2021). In addition to these functions, FAK modulates key cellular processes including proliferation, migration, and invasion (Dawson *et al.* 2021). Notably, FAK is capable of translocating into the nucleus, where it can regulate gene expression by interacting with transcriptional machinery, independently of its kinase activity (Schlaepfer *et al.* 2024). Although mutations in PTK2 are uncommon in human cancers (Sanchez-Vega *et al.* 2018), its amplification is more often detected in human cancer (Sulzmaier *et al.* 2014). Previous studies demonstrated that FAK overexpression confers pro-migratory and proliferative features to cancer cells through its kinase activity (Steinestel *et al.* 2020, Zhang *et al.* 2016). However, FAK has also been described to have a scaffold function that does not require its kinase activation (Lim *et al.* 2010); thus, it is important to study both kinase-related and scaffold roles of FAK.

To date, FAK’s role in GI-NET biology has not been systematically investigated. However, given its known interactions with pathways implicated in NET development (Garcia-Carbonero *et al.* 2023, Schlaepfer *et al.* 2024), it is possible that targeting FAK may offer new insights into the molecular vulnerabilities of these tumors.

Proteolysis targeting chimeras (PROTACs) are molecules designed to induce target protein degradation (Sulzmaier *et al.* 2014, Cromm *et al.* 2018, Popow *et al.* 2019). Using a PROTAC-FAK will block its kinase and scaffolding signaling activities. This study aims to investigate the impact of FAK inhibition on GI-NET cell biology to assess its potential as a therapeutic target in this setting. To this goal, we employed Y15, a classical kinase inhibitor, to block FAK activity, and PROTAC-FAK (BI-0319) to block its scaffold function. By combining pharmacological inhibition and genetic silencing, we aimed to evaluate the effects of FAK disruption on GI-NET cell viability, apoptosis, invasiveness, and transcriptional regulation, and to assess its potential as a therapeutic target in this challenging tumor type.

## Materials and methods

### Cell culture

GOT1 were kindly gifted by Ola Nilsson, Sahlgrenska Center of Cancer Research, University of Gothenburg, Sweden (Kölby *et al.* 2001, Hofving *et al.* 2018), COLO320DM were obtained from ATCC® CCL220™ and cultivated as previously described (Mygland *et al.* 2021). GOT1 were maintained in RPMI 1640 Medium (Sigma Aldrich, Italy) with 10% FBS (Thermo Fisher, Italy), penicillin/streptomycin, and insulin, transferrin, selenium solution (Thermo Fisher). COLO320DM were maintained in RPMI 1640 Medium (Sigma Aldrich) with 10% FBS (Thermo Fisher) and penicillin/streptomycin (Thermo Fisher). All cell lines used in the laboratory were maintained at low passage numbers and regularly authenticated based on viability, morphology, and growth characteristics. Routine mycoplasma testing was performed to ensure culture integrity.

### Compounds

Y15 was purchased from Sigma Aldrich, while PROTAC-FAK (BI-0319) was kindly provided by Boehringer Ingelheim via its open innovation platform opnMe (https://www.opnme.com).

### Antibodies and reagents

Rabbit anti-human p-ERK, total ERK, Cyclin A2, and H3K9ac antibodies were obtained from ABClonal Europe. Rabbit anti-human RB1 and rabbit anti-H3K4me antibodies were purchased from Abcam. Mouse anti-human GAPDH and rabbit anti-human FAK antibodies were purchased from Thermo Fisher. Hoechst 33342 was sourced from Sigma-Aldrich. ABflo® 647 mouse anti-rabbit IgG secondary antibody was also obtained from ABClonal Europe. IRDye® 800CW anti-rabbit IgG and IRDye® 680CW anti-mouse IgG secondary antibodies were purchased from LI-COR Biosciences.

### 2D and 3D cell viability assay

To assess cell viability in 2D culture, a crystal violet assay was conducted as previously described (Vella *et al.* 2024). Briefly, using low-serum medium, 3 × 10^3^ cells were seeded into each well of a 96-well plate. Twenty-four hours post-seeding, cells were treated with the indicated compounds for 72 h. Following treatment, cells were fixed for 20 min with 4% paraformaldehyde (PFA) and subsequently stained with 0.1% crystal violet solution. After overnight drying, the stain was solubilized using 10% acetic acid, and absorbance was measured using a Synergy BioTek plate reader.

To assess cell viability in 3D culture, the CellTiter-Glo® 3D Cell Viability Assay (Promega, Italy) was performed as previously described (Bresciani *et al.* 2019*a*, Gagliano *et al.* 2013). Cells were seeded at 2,000 cells per well in a 96-well BIOFLOAT™ 3D culture plate (faCellitate/Sarstedt, Germany) and centrifuged at 300 *g* for 1 min. Spheroid formation was allowed for 3 days before treatment with the indicated compounds. ATP levels, as an indicator of cell viability, were measured using the CellTiter-Glo® 3D assay according to the manufacturer’s instructions and measured using a Synergy BioTek plate reader.

### Colony formation assay

Colony formation was assessed as previously described (Vella *et al.* 2024). A total of 3 × 10^3^ GOT1 cells were seeded into 12-well plates and an equal number of COLO320DM were seeded into 6-well plates containing low-serum medium. Twenty-four hours post-seeding, cells were treated with the indicated compounds or vehicle control and incubated at 37°C for 14–20 days. The culture medium was refreshed every 3–4 days. Colonies were stained with crystal violet following the protocol described above and subsequently quantified and counted using ImageJ software.

### Caspase assay

The caspase assay was performed as previously described (Gagliano *et al.* 2015). Cells were seeded into 96-well plates and treated with the indicated compounds for 24 h. Caspase-3/7 activation was assessed using the Caspase-Glo® 3/7 Assay Kit (Promega), following the manufacturer’s instructions. Luminescence was measured using a Synergy BioTek plate reader.

### Invasion assay

The invasion assay was performed as previously described (Gagliano *et al.* 2020). Briefly, 8 μm pore size 24-well cell culture inserts (Sarstedt) were coated with Matrigel (Corning, USA). A total of 5 × 10^5^ cells were seeded into each insert using low-serum medium and treated with the indicated compounds. Medium containing 20% FBS was added to the lower chamber to serve as a chemoattractant and promote invasion. After 24 h, cells were fixed with 4% paraformaldehyde and stained with 0.1% crystal violet. Invasive cells on the lower surface of the membrane were counted under an inverted microscope.

### Scratch assay

At 24 h post-transfection, cells were seeded onto poly-L-lysine-coated 24-well plates in low-serum medium. After an additional 24 h incubation, a scratch wound was introduced into the cell monolayer using a sterile 200 μL pipette tip. Subsequently, the medium was replaced with complete growth medium, and images were captured immediately (time 0, T0). Plates were incubated for 96 h, after which images were acquired again (time 96 h, T96).

### Western blot

Western blot was performed as previously described (Gagliano *et al.* 2015). Briefly, cells were lysed using RIPA buffer supplemented with protease inhibitors as previously described (Stebbing *et al.* 2018). Protein concentrations were determined using a Protein Assay Kit (Thermo Fisher) according to the manufacturer’s instructions. Thirty micrograms of protein from each sample were separated on 4–20% SDS-PAGE gradient gels and transferred onto nitrocellulose membranes. Membranes were then incubated with the indicated primary antibodies, followed by appropriate fluorescent secondary antibodies. Protein bands were detected and visualized using the LI-COR Odyssey CLx imaging system. Densitometry was performed using ImageJ software (Supplementary Figure 2 (see section on Supplementary materials given at the end of the article)).

### Immunofluorescence

Immunofluorescence staining was used to assess FAK expression and was performed as previously described (Ditsiou *et al.* 2020). Cells were seeded onto sterile glass coverslips and allowed to adhere overnight. Following treatment with the indicated compounds, cells were fixed with 4% PFA for 20 min at room temperature, then permeabilized with 0.1% Triton X-100 in PBS for 10 min. After washing with PBS, cells were blocked with 5% bovine serum albumin in PBS for 1 h to prevent non-specific binding. Primary staining was carried out using rabbit anti-human FAK antibody (Thermo Fisher) diluted in blocking buffer and incubated overnight at room temperature. After washing, cells were incubated with ABflo® 647-conjugated mouse anti-rabbit IgG secondary antibody (ABClonal Europe, Germany) and Hoechst 33342 (Sigma-Aldrich) for nuclei staining for 1 h at room temperature in the darkness. Coverslips were then mounted on glass slides using anti-fade mounting medium. Images were acquired using a confocal microscope (LEICA LSM 880), and FAK staining was analyzed using LEICA software. Fluorescence intensity was measured using ImageJ software (Supplementary Figure 2) .

### RNA extraction and qPCR

Total RNA was extracted using the PureLink™ RNA Mini Kit (Invitrogen, Italy), following the manufacturer’s instructions. RNA concentration and purity were assessed using a NanoDrop 2000 spectrophotometer (Thermo Fisher). Complementary DNA (cDNA) was synthesized using the High-Capacity cDNA Reverse Transcription Kit (Thermo Fisher) according to the manufacturer’s protocol. Quantitative real-time PCR (qRT-PCR) was performed using SYBR Green-based gene expression assays (Thermo Fisher). Samples were run on a QuantStudio 1™ thermal cycler (Applied Biosystems, Italy) and analyzed with DA2 software (Applied Biosystems). Each experiment included at least *n* = 2 biological replicates and *n* = 3 technical replicates. GAPDH was used as the internal control. Primer sequences used in qPCR are listed in Table 1.

**Table 1:** qPCR primers.

Gene	Primer	Sequence
PTK2	Forward	GTA​TGT​CCC​TAT​GGT​GAA​GG
PTK2	Reverse	GGT​CAG​AGT​TCA​ATA​GCT​TC
GAPDH	Forward	TCGGAGTCAACGGATTTG
GAPDH	Reverse	CAA​CAA​TAT​CCA​CTT​TAC​CAG​AG
RB1	Forward	CAG​AAA​TGA​CTT​CTA​CTC​GAA​C
RB1	Reverse	AAT​GTG​GCC​ATA​AAC​AGA​AC

### esiRNA transfection

Mission esiRNA targeting human PTK2 (FAK) EHU077321 and negative control were purchased from Sigma-Aldrich. Oligos were transfected using HiPerFect Transfection Reagent (QIAGEN), as previously described (Gagliano *et al.* 2020) following the manufacturer’s instructions. FAK silencing was evaluated by qPCR (Supplementary Figure 1).

### Tumor gene expression datasets

Gene expression data (raw counts) and clinical information of 212 gastro-entero-pancreatic neuroendocrine tumors were downloaded from GEO (GSE98894) and analyzed using code produced and adjusted by GEO2R and the R package GEOquery (version 2.74.0). Principal component analysis was performed using the R package DESeq2 (version 1.46.0) and the function plotPCA after batch effect correction performed using the R package sva (version 3.54.0). The beeswarm and scatter plots in Fig. 5A and B were obtained using the R packages ggplot2 (version 3.5.1), ggbeeswarm (version 0.7.2), ggpubr (version 0.6.0), and ggrepel (version 0.9.6). Correlations shown in Fig. 5B were calculated using the inbuilt R function *cor.test*.

### Statistical analysis

Statistical analyses were performed using GraphPad Prism 10 software. Depending on the specific experiment, appropriate statistical tests were applied and are indicated in the respective figure legends. A *P*-value <0.05 was considered statistically significant. Unless otherwise specified, experiments were carried out using three independent biological replicates.

## Results

### FAK inhibition reduces cell viability and increases apoptosis in GI-NET cell lines

**Figure 1:**
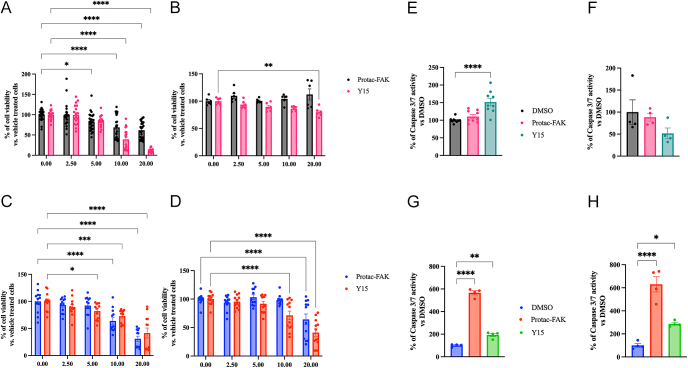
Effects of FAK inhibition on GI-NETs cells in 2D and 3D culture. COLO320DM and GOT1 cells were seeded in 96-well plates and incubated for 72 h with increasing concentrations of PROTAC-FAK and Y15, control cells were treated with a vehicle (DMSO). Cell viability was assessed for 2D COLO320DM (A) and 2D (C) GOT1 was assessed by crystal violet assay in three independent experiments with at least six replicates each, and it is expressed as the mean ± SEM. *P < 0.05 vs vehicle cells; ***P < 0.0001 vs vehicle cells. ****P < 0.00001 vs vehicle cells. Cell viability of 3D COLO320DM (B) and 3D GOT1 (D) was measured as luminescent output in three independent experiments with at least two replicates each, and it is expressed as the mean ± SEM **P < 0.01 vs vehicle cells, ****P < 0.00001 vs vehicle cells. Significance was calculated by 2-way ANOVA using Dunnett’s multiple comparison test. Caspase activity of 2D (E) and 3D (F) COLO320DM and 2D (G) and 3D (H) GOT1 was measured as luminescent output in two independent experiments with two replicates each, and it is expressed as the mean ± SEM. ***P < 0.01 vs vehicle cells; ****P < 0.00001 vs vehicle cells. Significance was calculated by 2-way ANOVA using Dunnett’s multiple comparison test.

To assess the impact of FAK inhibition on the viability of GI-NET cells, COLO320DM and GOT1 cells were treated with increasing concentrations of PROTAC-FAK and Y15 (0–20 μM range). In 2D cultures, a significant dose-dependent reduction in cell viability was observed in both COLO320DM (Fig. 1A) and GOT1 (Fig. 1C) cells, as assessed by crystal violet assay. A 10 μM concentration of both compounds was enough to significantly reduce cell viability. In 3D spheroid cultures of COLO320DM (Fig. 1B) and GOT1 (Fig. 1D), treatment with FAK inhibitors decreased their efficacy in reducing cell viability. While in GOT1 3D culture, higher concentrations of both compounds (20 μM) were still effective, in COLO320DM only treatment with Y15 20 μM was able to reduce cell viability (Fig. 1B).

Increased caspase activity confirmed apoptosis induction following FAK inhibition. Both 2D (Fig. 1G) and 3D (Fig. 1H) cultures of GOT1 exhibited significantly elevated caspase luminescence following treatment, with more pronounced effects upon treatment with PROTAC-FAK. In COLO320DM 2D (Fig. 1E), only Y15 was able to induce caspase 3/7 activation, while this effect was not detectable in 3D (Fig. 1F). The results suggest that FAK inhibition triggers apoptotic pathways, contributing at least partially to the observed reduction in cell viability.

### FAK inhibition impairs clonogenicity and invasion of GI-NET cells

**Figure 2:**
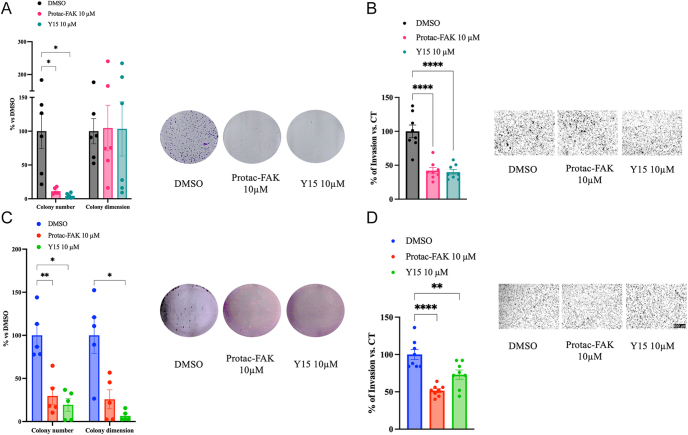
FAK inhibitors decrease clonogenic and invasive potential of GI-NETs. FAK inhibition decreased colony formation and invasion potential in GI-NET cell lines. Changes in colony number and dimension of COLO320DM (A) and GOT1 (C) after treatment with PROTAC-FAK and Y15, with representative images of colony formation assays. Colonies were quantified using ImageJ software, and results show the percentage of colonies formed after treatment with the indicated concentrations of the drug (surviving fraction), corrected according to the plating efficiencies of the corresponding controls. Data are shown as mean ± SEM of at least three independent experiments. *P < 0.05 vs vehicle cells. Significance was calculated by 2-way ANOVA using Sidak’s multiple comparison test. For the invasion assay, COLO320DM (B) and GOT1 (D) cells were seeded on the Matrigel-coated upper chamber of the transwell. Twenty-four hours later, migrated cells were fixed, stained, and counted (*n* = 3 independent experiments, minimum two technical replicates). Significance was calculated using 1-way ANOVA. Data are expressed as mean ± SEM; **P < 0.01, ****P < 0.0001 vs DMSO. Significance was calculated by ANOVA using Dunnett’s multiple comparison test.

We next examined the effect of FAK inhibition on colony-forming potential. Both COLO320DM (Fig. 2A) and GOT1 (Fig. 2C) clonogenic potential was affected by FAK inhibitors. In GOT1, a significant reduction in the number and size of colonies was observed upon treatment with either PROTAC-FAK or Y15. Quantification of colonies revealed a decline in surviving fractions upon blocking FAK. Invasion assays demonstrated that treatment with PROTAC-FAK and Y15 markedly reduced the invasive capacity of COLO320DM (Fig. 2B) and GOT1 (Fig. 2D) cells compared to DMSO-treated controls, indicating that FAK activity is critical for maintaining the invasive properties of GI-NET cells.

### FAK inhibition modulates signaling pathways and gene expression

**Figure 3:**
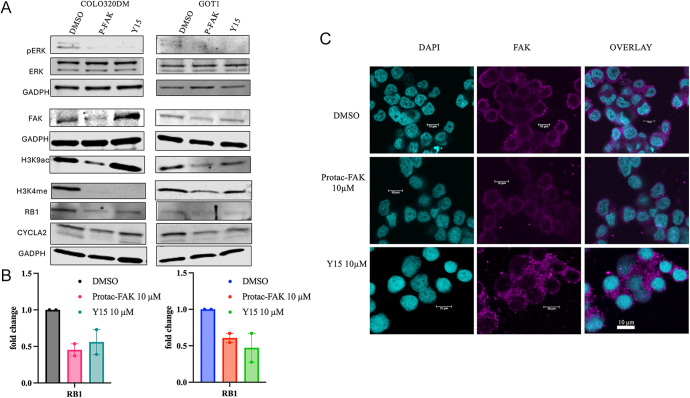
MAPK and epigenetic signaling are impaired by FAK inhibition. Effects of FAK inhibition in cell protein expression and signaling. (A) Western blotting of total and phosphorylated ERK1/2, FAK, RB1, Cyclin A2, H3K9-acetylated, and H3K4-methylated after treatment with PROTAC-FAK and Y15 10 μM. GAPDH was used as a loading control. (B) qRT-PCR of RB1 expression levels in GI-NET cells following treatment with PROTAC-FAK and Y15 10 μM. (C) Representative images of immunofluorescent staining of COLO320DM cells for FAK after treatment with PROTAC-FAK and Y15 10 μM. Original magnification: ×60.

To investigate the molecular mechanisms underlying the effects of FAK inhibition, Western blot analyses were performed. Both PROTAC-FAK and Y15 treatments led to decreased levels of phosphorylated ERK1/2, while total protein levels remained unchanged. On the other hand, as expected, only treatment with PROTAC-FAK reduced FAK protein levels (Fig. 3A central panel). Notably, H3K9 acetylation was also reduced, but only by PROTAC-FAK, suggesting that reduced levels of FAK can affect epigenetic modulation and thus gene expression (Fig. 3A, central panel). The involvement of FAK in epigenetic mechanisms was further supported by the observed reduction in H3K4 methylation following treatment with FAK inhibitors (Fig. 3A, lower panel). The decrease in Cyclin A2 levels (Fig. 3A, lower panel) upon FAK inhibition further supports a role for FAK in regulating cell cycle progression.

Western blot and qRT-PCR analysis showed increased RB1 mRNA expression following FAK inhibition (Fig. 3A lower panel and 3B), and immunofluorescence imaging confirmed a reduction in FAK expression in COLO320DM cells post-treatment with PROTAC-FAK (Fig. 3C), supporting effective FAK targeting.

### FAK silencing recapitulates the effects of pharmacological inhibition

**Figure 4:**
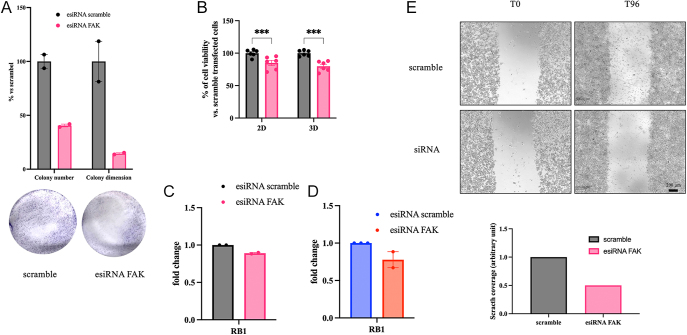
FAK silencing recapitulates the effects of FAK inhibitors. FAK silencing reduced colony formation, viability, mobility, and RB1 gene expression in GI-NET cell lines. Changes in colony number and dimension of COLO320DM (A) after silencing of FAK with esiRNA, with representative images of colony formation assays. Colonies were quantified using ImageJ software and results show the percentage of colonies formed after treatment with the indicated concentrations of the drug (surviving fraction), corrected according to the plating efficiencies of the corresponding controls. (B) Cell viability was assessed in 2D and 3D COLO320DM was measured as luminescent output in three independent experiments with at least two replicates each, and it is expressed as the mean ± SEM **P < 0.01 vs vehicle cells, ***P < 0.0001 vs scramble-transfected cells. Significance was calculated by 2-way ANOVA using Sidak’s multiple comparison test. qRT-PCR of RB1 expression levels in COLO320DM (C) and GOT1 (D) cells following transfection with FAK esiRNA. (E) COLO320DM transfected with scramble or FAK targeting esiRNA were plated on a poly-L-lysine-coated 24-well plate well and allowed to attach overnight. Wells were then wounded with a p20 pipette tip and imaged immediately and after 96 hours.

To validate the role of FAK in GI-NET cell proliferation and gene expression, esiRNA-mediated silencing of FAK was performed. Colony formation was significantly impaired in COLO320DM cells transfected with FAK esiRNA compared to scramble controls (Fig. 4A). Cell viability assays in both 2D and 3D conditions confirmed reduced proliferation (Fig. 4B), consistent with pharmacological inhibition results.

FAK knockdown also downregulates RB1 expression in both COLO320DM (Fig. 4C) and GOT1 (Fig. 4D) cells. Furthermore, wound-healing assays demonstrated a substantial reduction in cell motility in FAK-silenced cells at 96 h compared to controls (Fig. 4E), reinforcing the role of FAK in GI-NET cell migration and survival.

### FAK (PTK2) gene expression is highly expressed in rectal and small intestine GI-NETs and positively correlates with RB1 expression

**Figure 5:**
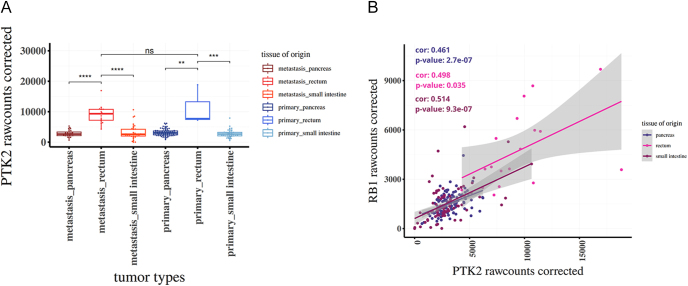
FAK and RB1 interplay is confirmed by patient DATA sets. (A) Boxplot showing PTK2 (FAK) expression levels (corrected raw counts) across various GI-NET tumor types and tissue origins, including metastatic and primary samples from the pancreas, rectum, and small intestine. Significant differences were observed among multiple groups, with notably higher PTK2 expression in metastatic and primary tissues coming from rectal primary sites compared to other primary tumors and metastasis sites. (B) Scatter plot showing a positive correlation between PTK2 and RB1 expression across GI-NET samples, stratified by tissue origin. Correlation coefficients (Pearson’s r) and *P*-values are indicated for each tissue type: pancreas (*r* = 0.461, *P* = 2.7e-07), rectum (*r* = 0.498, *P* = 0.035), and small intestine (*r* = 0.514, *P* = 9.3e-07). Linear regression lines with 95% confidence intervals are shown.

To explore the clinical relevance of FAK (PTK2) expression in GI-NETs, we analyzed PTK2 gene levels across different tumor types and tissue origins using publicly available datasets (Alvarez *et al.* 2018) (GSE98894). PTK2 (FAK) expression was significantly higher in rectal NETs compared to pancreatic and small intestinal NETs (Fig. 5A). Further analysis revealed a significant positive correlation between PTK2 and RB1 gene expression across all tissues. Correlation was strongest in small intestinal NETs (*r* = 0.514, *P* = 9.3e-07), followed by rectal (*r* = 0.498, *P* = 0.035) and pancreatic tumors (*r* = 0.461, *P* = 2.7e-07) (Fig. 5B). These data support a potential transcriptional link between FAK signaling and RB1 expression in GI-NETs, aligning with our *in vitro* findings.

## Discussion

**Figure 6:**
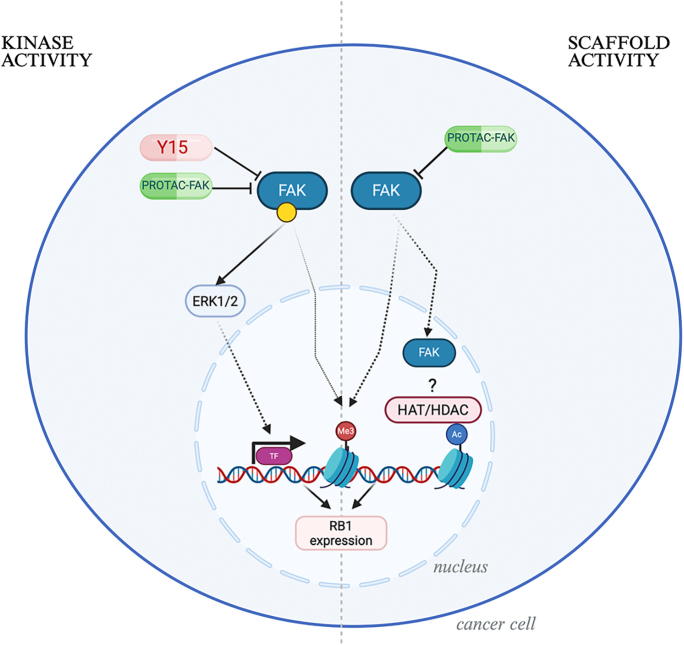
Dual role of FAK in GI-NETs. Schematic model illustrating the dual role of FAK in gastrointestinal neuroendocrine tumors (GI-NETs). FAK regulates oncogenic pathways through both its kinase-dependent and scaffold (kinase-independent) functions. On the left, Y15 and PROTAC-FAK inhibit the kinase activity of FAK, leading to reduced ERK1/2 signaling and downstream suppression of RB1 transcription. On the right, PROTAC-FAK uniquely targets the scaffold function of FAK, which includes its nuclear translocation and putative interaction with histone acetyltransferases (HATs) or histone deacetylases (HDACs). This interaction may alter chromatin accessibility and modulate epigenetic control of RB1 expression, independent of classical phosphorylation signaling. This model supports a dual mechanism by which FAK promotes GI-NET cell survival and gene regulation. Created in BioRender. Gagliano T (2025) (https://BioRender.com/ddyjxrf).

Our findings demonstrate that FAK is a critical regulator of GI-NET cell survival, invasiveness, and gene expression. Both pharmacological inhibition and genetic silencing of FAK led to reduced cell viability, impaired clonogenicity, and increased apoptosis in GI-NET cell lines, highlighting its functional importance in tumor progression. Furthermore, FAK inhibition suppressed ERK1/2 phosphorylation in response to both Y15 and PROTAC-FAK treatment, confirming disruption of canonical MAPK signaling downstream of FAK (Zhang *et al.* 2016, Steinestel *et al.* 2020). Despite ERK1/2 activation has been described to regulate histone modification, only PROTAC-FAK treatment led to a reduction in H3K9 acetylation levels, indicating that FAK’s role in epigenetic regulation is likely scaffold-dependent and independent of its kinase activity (Sun *et al.* 2024). Furthermore, FAK has been reported to regulate chromatin methylation (Jeong *et al.* 2021). Consistent with this, our results showed that methylation levels of H3K4 were decreased upon FAK inhibition. These findings support the emerging concept that nuclear FAK may contribute to chromatin remodeling and transcriptional control through non-catalytic interactions with the epigenetic machinery (Schlaepfer *et al.* 2024). FAK has also been reported in other contexts to regulate the expression of RB1 (Zhang *et al.* 2023). The observed upregulation of *RB1* expression following FAK inhibition points to a potential link between FAK activity and the transcriptional regulation of cell cycle genes. Although *RB1* overexpression is not typically oncogenic, dysregulation of RB1 activity – particularly in the context of imbalances with other checkpoint regulators such as CDKs and p53 – could disrupt cell cycle control in a manner that promotes tumorigenesis (Zhao *et al.* 1998, Engeland 2022). This raises the possibility that FAK-mediated signaling may sustain an altered transcriptional program that supports GI-NET survival and proliferation. The dual inhibition strategy employed in this study – targeting both kinase-dependent and scaffold-mediated functions of FAK – proved especially informative. Treatment with PROTAC-FAK had a more pronounced impact on apoptosis and epigenetic modulation than kinase inhibition alone, supporting the hypothesis that FAK’s non-catalytic functions contribute substantially to its oncogenic role (Fig. 6). These findings align with the recent literature suggesting that nuclear FAK can participate in chromatin remodeling and influence gene transcription independently of its enzymatic activity (Lim 2013, Dawson *et al.* 2020, Sato *et al.* 2020, Griffith *et al.* 2021). The differential responses observed in 2D versus 3D cultures further emphasize the complexity of tumor architecture in shaping therapeutic outcomes. In 3D spheroid models, which better recapitulate the *in vivo* setting, higher concentrations of FAK inhibitors were required to achieve comparable effects, particularly in COLO320DM cells. These differences highlight the importance of incorporating 3D culture systems in preclinical evaluations of drug efficacy, and suggest that tumor context and cellular organization may influence FAK inhibitor sensitivity (Bresciani *et al.* 2019*b*). Interestingly, while both inhibitors impaired colony formation and invasion, they did not significantly alter colony size in COLO320DM cells, suggesting that FAK inhibition primarily affects the ability of single cells to initiate new colonies rather than limiting the expansion of already proliferating clones. This phenotype may reflect FAK’s role in early-stage anchorage-independent growth, cell adhesion, and survival signaling – critical steps in metastatic colonization (Murphy *et al.* 2020). At the molecular level, the observed decrease in ERK1/2 phosphorylation aligns with previous studies demonstrating that FAK acts upstream of MAPK signaling pathways to promote cell proliferation and survival (Zhang *et al.* 2016, Steinestel *et al.* 2020). The reduction in H3K9 acetylation upon FAK inhibition adds to a growing body of evidence indicating that FAK plays a role in epigenetic regulation (Sun *et al.* 2024). Acetylation of histone H3 at lysine 9 is associated with active transcription, and its suppression suggests that FAK may be involved in maintaining an open chromatin state conducive to gene expression. This potential mechanism is further supported by the decrease in histone methylation observed following treatment with FAK inhibitors. As FAK has been described to modulate RB1 expression (Zhang *et al.* 2023), we decided to investigate changes in the expression of this gene upon treatment with FAK inhibitors or after FAK silencing. Our data showing modulation of *RB1* expression further support a model in which FAK participates in transcriptional regulation beyond its classical cytoplasmic signaling role. To validate these findings and confirm the specificity of the observed effects, we employed esiRNA-mediated silencing of *PTK2* (FAK), which recapitulated the phenotypic and molecular alterations seen with pharmacological inhibition. Reduced colony formation, viability, and cell motility upon FAK knockdown reinforce the idea that FAK is essential for the maintenance of oncogenic properties in GI-NETs. The clinical relevance of these findings is further underscored by our in silico analysis of publicly available transcriptomic datasets, which revealed that *PTK2* is highly expressed in rectal and small intestinal NETs compared to pancreatic NETs. Importantly, *PTK2* expression positively correlated with *RB1* levels across all tumor types, suggesting a consistent transcriptional relationship that may reflect a broader, tissue-independent mechanism of regulation (Alvarez *et al.* 2018). Despite the promising results, further analysis has to be performed in order to clarify the exact involvement of FAK in the epigenetic machinery. In addition, while COLO320DM cells have been widely used in NET research (Iida *et al.* 2012, Narayan *et al.* 2019, Rodríguez-Remírez *et al.* 2020), they are not the ideal model for studying neuroendocrine tumors. Further validation of our findings using animal models or patient-derived cells will be necessary to confirm and extend these results. In summary, our findings suggest that FAK plays a pivotal role in the pathogenesis of GI-NETs by regulating survival, invasion, and gene expression. However, further investigations are required to comprehensively elucidate the mechanistic contributions of FAK, particularly its scaffold functions, in neuroendocrine tumor biology.

## Supplementary materials



## Declaration of interest

The authors declare that there is no conflict of interest that could be perceived as prejudicing the impartiality of the work reported.

## Funding

This work was supported by the Departmental Strategic Plan (PSD) of the University of Udine-Interdepartmental Project on Healthy Ageing (2020-25).

## Author contribution statement

TG was responsible for conceptualization, funding acquisition, supervision, visualization and writing original draft. LTo was responsible for data curation, investigation, validation, writing original draft and review and editing. VH and EM participated in the investigation. FD contributed to methodology and visualization. LTr was responsible for software, writing original draft and review and editing. AD contributed to writing original draft and writing review and editing.

## Ethics

This project has received approval from the Ethics Committee of the Department of Medicine at the University of Udine.
