# Supplementary material for: FAK inhibition disrupts tumor growth, apoptosis, and transcriptional regulation in GI-NETs
Source: Endocr Oncol. 2025 Aug 14;5(1):e250052. doi: 10.1530/EO-25-0052 (PMC12358824; doi:10.1530/EO-25-0052)

Supplementary figure 1.

qRT-PCR of FAK expression levels in COLO320DM (A) and GOT1 (B) cells following transfection FAK esiRNA.

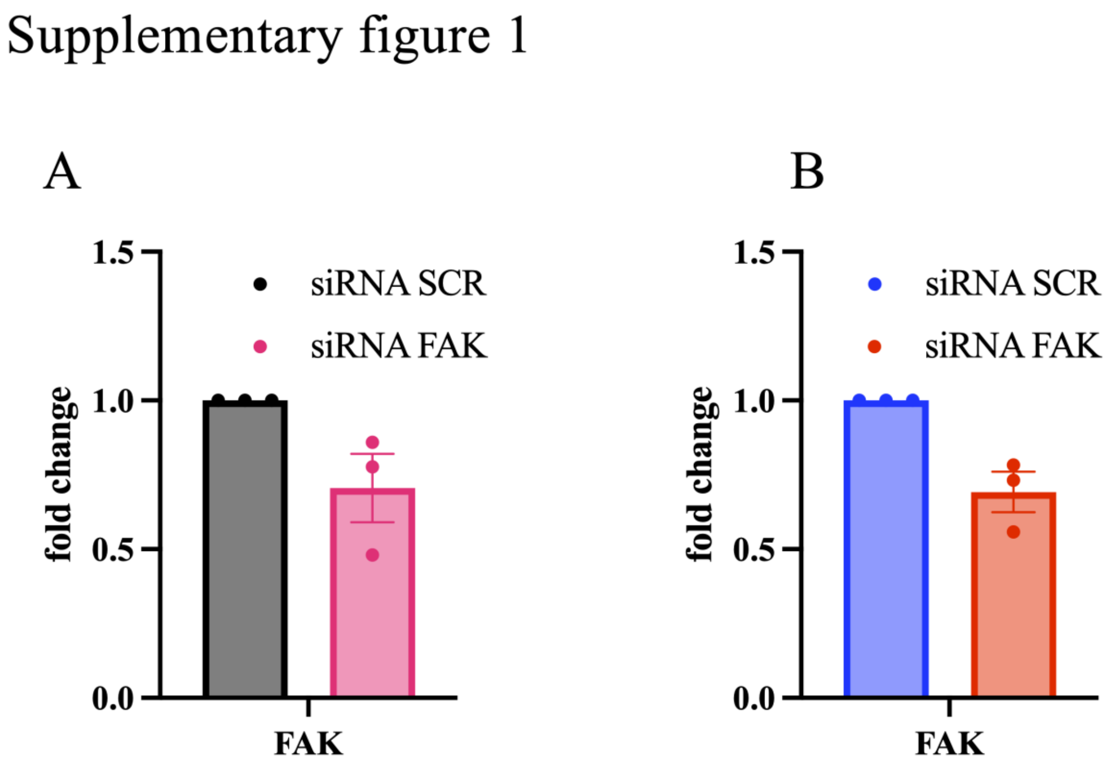

Supplementary figure 2

Densitometry of protein expression in COLO320DM (A) and GOT1 (B). FAK fluorescence intensity quantification in COLO320DM (C).

Supplementary figure 2

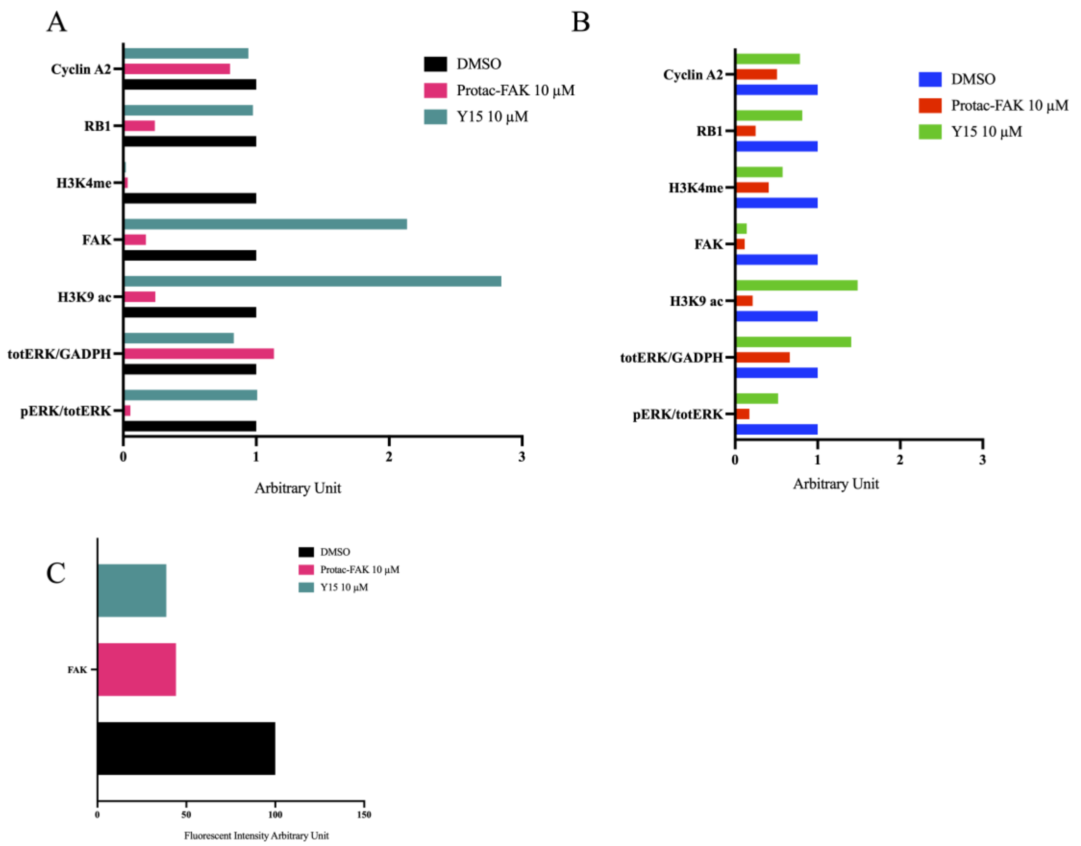

Supplement: Supplementary file 1 [file supplementary_materials.pdf]
